# Surface Reconstruction Assessment in Photogrammetric Applications

**DOI:** 10.3390/s20205863

**Published:** 2020-10-16

**Authors:** Erica Nocerino, Elisavet Konstantina Stathopoulou, Simone Rigon, Fabio Remondino

**Affiliations:** 1 LIS UMR 7020, Aix-Marseille Université, CNRS, ENSAM, Université De Toulon, Domaine Universitaire de Saint-Jérôme, Bâtiment Polytech, Avenue Escadrille Normandie-Niemen, 13397 Marseille, France; 2 3D Optical Metrology (3DOM) Unit, Bruno Kessler Foundation (FBK), 38123 Trento, Italy; estathopoulou@fbk.eu (E.K.S.); srigon@fbk.eu (S.R.); remondino@fbk.eu (F.R.); 3 Laboratory of Photogrammetry, National Technical University of Athens (NTUA), 15780 Athens, Greece

**Keywords:** surface reconstruction, mesh model, 3D reconstruction, visibility constraints, volumetric methods, dense point cloud, multiple view stereo (MVS), dense image matching (DIM), photogrammetry, computer vision

## Abstract

The image-based 3D reconstruction pipeline aims to generate complete digital representations of the recorded scene, often in the form of 3D surfaces. These surfaces or mesh models are required to be highly detailed as well as accurate enough, especially for metric applications. Surface generation can be considered as a problem integrated in the complete 3D reconstruction workflow and thus visibility information (pixel similarity and image orientation) is leveraged in the meshing procedure contributing to an optimal photo-consistent mesh. Other methods tackle the problem as an independent and subsequent step, generating a mesh model starting from a dense 3D point cloud or even using depth maps, discarding input image information. Out of the vast number of approaches for 3D surface generation, in this study, we considered three state of the art methods. Experiments were performed on benchmark and proprietary datasets of varying nature, scale, shape, image resolution and network designs. Several evaluation metrics were introduced and considered to present qualitative and quantitative assessment of the results.

## 1. Introduction

The 3D reconstruction of the physical shape or geometry of either single objects or complex scenes is a topic of interest in countless application scenarios, varying from more industrial analyses [1], cultural heritage related studies [2,3], environmental mapping [4,5] and city modeling [6,7] to the latest autonomous driving and navigation applications [8]. Polygonal meshes in the form of triangular or quadrilateral faces are typically used to represent the digital surface of such objects or scenes in the 3D space.

The employed technique used to acquire the input data highly affects the quality of the final surface reconstruction. Among a large variety of active and passive optical sensors and methods, image-based 3D reconstruction is frequently used due to its easiness, portability, efficiency and reliability. In particular, dense image matching (DIM) is the process of calculating the 3D coordinates of each pixel visible in at least two images, thus generating a dense representation of the scene. In photogrammetry, DIM follows the image orientation, triangulation and camera calibration steps commonly calculated within the bundle adjustment (BA) process [9].

Equivalent to this, in the computer vision community, the task of reconstructing a dense 3D representation of the scene from a collection of images is known as multi-view stereo (MVS) [10], typically performed as a subsequent step to the Structure from Motion (SfM) procedure.

Traditionally, the final output of DIM is a 3D point cloud (or, in mapping applications, 2.5D digital elevation model, DEM) of the scene [9,11]. The surface or mesh reconstruction is usually applied to the point cloud resulting from the DIM, without any further checks on the images and their orientations.

On the other hand, MVS encompasses distinct 3D reconstruction methods that may deliver different output products in the form of depth maps, point clouds, volume scalar-fields and meshes [12]. Such different scene representations have been initially developed for visualization and graphics applications, each of them being optimized for different purposes, following also the evolution in hardware and computational power. A certain class of MVS approaches generates a refined mesh model using photo-consistency (i.e., pixel color similarity) measures and the so-called visibility information (i.e., the image orientations and thus the 2D–3D projections) [12,13].

### Paper’s Motivation and Aim

This study aimed to investigate the surface reconstruction problem for image-based 3D reconstruction scenarios. The paper builds upon the following considerations:

- In a traditional photogrammetric pipeline, the meshing step interpolates a surface over the input 3D points. This is usually disjointed from the 3D point cloud generation DIM but can potentially leverage and take advantage of additional information from the previous steps of the workflow, i.e., visibility constraints and photo-consistency measures which are generally not considered in popular meshing algorithms as Poisson [14].
- Dense point clouds can be heavily affected by poor image quality or textureless areas, resulting in high frequency noise, holes and uneven point density. These issues can be propagated during the mesh generation process.
- Volumetric approaches for surface reconstruction based on depth maps are well-established, time-efficient methods for depth sensors, also known as RGB-D [15], and might be a valid approach also for pure image-based approaches.

**Figure 1:**
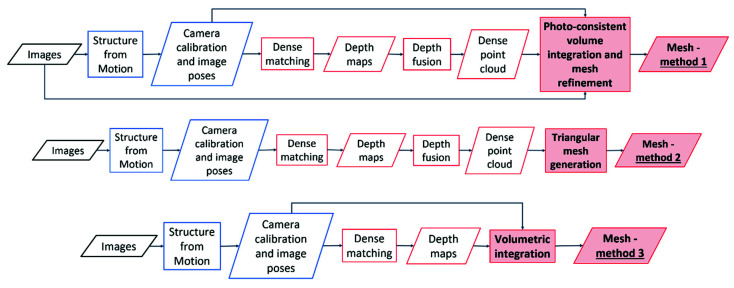
The three surface generation approaches investigated in the paper.

The aim of this work was thus to evaluate whether the integration of visibility information (image orientation) and photo-consistency and during the meshing process can potentially lead to an improvement of the mesh quality (and successive products). For this reason, three diverse surface reconstruction approaches were considered and evaluated on diverse datasets (Figure 1):

- *Method 1*: Surface generation and refinement are incorporated in the 3D reconstruction pipeline. The mesh is generated after depth maps and dense point clouds are estimated and is subsequently refined considering visibility information (i.e., image orientation) to optimize a photo-consistency score over the reconstructed surface [13,16].
- *Method 2*: Surface generation is disjoint from the image-based 3D reconstruction procedure. The dense point cloud, as obtained from Method 1, is converted to a mesh model without the use of any visibility constraints or photo-consistency checks [14,17].
- *Method 3*: Given the image poses, a mesh model is generated from the depth maps produced in Method 1, employing a volume integration approach [15,18,19]. Again, in this method, visibility and photo consistency information are not taken into consideration while reconstructing the surface.

The results of the considered approaches were evaluated using several metrics, including accuracy, completeness and roughness. On the contrary, the computational time was not considered a key factor for this investigation.

The rest of the article is divided as follows. Section 2 reviews the main concepts and steps of DIM and MVS. Section 3 provides an overview of the available DIM/MVS benchmark datasets, examining their suitability for the present study; surface reconstruction and assessment criteria are also addressed. The considered surface reconstruction methods are then introduced in Section 4. The employed datasets, carefully chosen to cover a wide range of image scale, image resolution and application scenarios (from close range to aerial photogrammetry), and the adopted comparative metrics are presented in Section 5, followed by a discussion of the obtained results in Section 6.

## 2. On DIM and MVS

Matching is a general term used to define approaches for finding correspondences between two images, sets of features or surfaces [20]. In photogrammetry, image matching indicates the (automatic) procedure of identifying and uniquely matching corresponding (homologous, conjugate) features (points, patterns and edges) between two (i.e., stereo) or more (i.e., multi-view) overlapping images. In computer vision, the analogous step is the so-called “stereo correspondence” problem [21].

Image matching can be sparse or dense, stereo or multi-view. In sparse matching, detectors and descriptors are usually employed to extract and characterize a set of sparse and potentially matching image features; their local appearance is then used to search and match corresponding locations in other images. Some approaches first extract only highly reliable features and then use them as seeds to grow additional matches [10]. Sparse matching algorithms are an integral part of automatic image orientation procedures implemented in SfM algorithms.

In dense image matching (DIM), a huge number of correspondences (up to pixel-to-pixel) between image pairs (dense stereo matching) or multiple views is established. The dense correspondence problem is still a crucial and active research topic for applications where dense and detailed 3D data generation are needed. It is more challenging than the sparse correspondence problem, since it requires inferring correspondences also in textureless, reflective and challenging areas [10]. Szeliski [10] identified four main steps that are usually implemented in dense correspondence algorithms: (1) matching cost computation; (2) cost (support) aggregation; (3) disparity computation and optimization; and (4) disparity refinement. Based on the various implementations of the aforementioned fundamental steps, diverse methods have been proposed. Several approaches have been developed to measure the agreement between the pixels and find the best match, from local to semi-global [22] and global methods, from area or patch-based [23,24] to feature-based [25] or a combination of them [26]. The most important used criterion to find corresponding pixels is known as photo-consistency, which estimates the similarity of two (or more) pixels between two images [12]. Examples of photo-consistency metrics are the Sum of Squared Differences (SSD), Sum of Absolute Differences (SAD), Normalized Cross Correlation (NCC) and Mutual Information (MI) [12].

The term dense stereo matching refers to the subclass of dense correspondence methods focusing on establishing correspondences between pixels in a stereo pair of images [20]. When three or more overlapping images are involved in the reconstruction process, the dense matching problem is defined as multi-view, multi-view stereo or multiple view. The ultimate goal of MVS is to reconstruct a complete and potentially globally consistent 3D representation of the scene from a collection of images acquired from known positions [10,27].

Examples of MVS algorithms are surface-based stereo, voxel coloring, depth map merging, level set evolution, silhouette and stereo fusion, multi-view image matching, volumetric graph cut and carved visual hulls [10]. An exhaustive taxonomy of DIM and MVS methods is extremely complex and, thus, several classification schemes have been proposed up to now. For instance, Seitz et al. [27] divided MVS algorithms based on six criteria: scene representation, photo-consistency measure, visibility model, shape prior, reconstruction algorithm and initialization requirements. Aanæs et al. [28] divided MVS approaches into two main categories: point cloud based methods (e.g., [23,29,30,31,32]) and volume-based methods (e.g., [33,34,35]).

In this paper, we adapt the categorization proposed by Furukawa and Hernández [12], focusing on the different output of the MVS procedure in terms of scene’s representation. MVS starts with the search of corresponding pixels in the images in order to transform these dense correspondences into depth maps and/or point clouds. Visibility and occlusions estimation can be integrated in the matching process and are usually performed in coarse-to-fine manner as the dense reconstruction progresses to optimize the photo-consistency computation [12]. Based on the photo-consistency result (i.e., once the corresponding pixels have been identified in the images), depth maps are reconstructed for each image used as reference and matched with its visual neighbors. The resulting depth maps are then merged to produce the final 3D point cloud. Alternatively, when corresponding pixels with the highest photo-consistency score are found in two or more images, they are directly converted into 3D coordinates using collinearity in order to generate a dense point cloud.

Subsequently, the mesh generation follows. Oriented (i.e., with normals) and unoriented (i.e., without normals) point clouds can be converted into mesh models using several algorithms, such as Poisson surface reconstruction [14]. Alternatively, more sophisticated optimization techniques or volumetric surface reconstruction approaches have also been largely investigated [13]. Some of them require dense point clouds as an intermediate step while generating the surface model [36,37]. Other volumetric methods, such as the so-called Truncated Signed Distance Field algorithm (TSDF), use straightaway depth maps and generate a surface by dividing the 3D space into 3D voxel cells where each voxel is labeled with a distance [38]. However, while surface reconstruction from depth maps is quite common when using RGB-D sensors [15,39], in image-based 3D applications of metric accuracy it is still not fully exploited. Indeed, point clouds are the most common and requested product of a photogrammetric project while mesh models are generally produced mainly for rendering and visualization purposes.

Finally, a mesh refinement step can be undertaken [12]. This requires that images are considered again to verify the photo-consistency, this time over the reconstructed mesh surface. The vertices are moved to optimize their location, individually or all together. In the optimization process, a regularization term can influence the smoothness of the final mesh and, when available, silhouettes can be included as an additional consistency measure.

## 3. Benchmarks and Assessment of Surface Reconstruction Approaches

The current section is divided in two parts. In the first, existing benchmarks and evaluation methods adopted in photogrammetry and computer vision are reviewed, showing that they mainly focus on dense point clouds. The second part addresses the quality metrics developed in computer graphics for the assessment of surface reconstruction approaches. Some of these metrics were adopted in the comparative evaluation presented in Section 6.

The use of benchmarks is a common practice in the scientific community for the purpose of assessing and comparing different techniques, methods and algorithms. They collect data characterized by relevant features and evaluated according to significant metrics. A benchmark is usually composed of three elements: (1) input data to apply the investigated method; (2) reference or ground truth data against which the achieved results are compared; and (3) assessment criteria for the evaluation procedure.

### 3.1. DIM/MVS Benchmarks

Bakuła et al. [40] and Özdemir et al. [41] provided overviews of benchmarking initiatives proposed in photogrammetry and remote sensing. Knapitsch et al. [42] and Schops et al. [43] discussed and proposed benchmarks specifically focusing on MVS. Each of the available benchmarks has unique features, which cover different scene characteristics, from small objects in laboratory conditions, such as in Middlebury MVS [27,44] and DTU Robot Image Data Sets [28,45] or 3DOMcity benchmark [41,46], to more and complex scenes both indoor and outdoor (Strecha [47], ETH3D [43,48]; Tanks and Temples [42,49]; ISPRS-EuroSDR benchmark on High Density Aerial Image Matching [50,51]. Image resolutions also vary, from very small (0.2 Mpx Middlebury MVS) to medium (6 Mpx Strecha) up to high (24 Mpx 3DOMcity and ETH3D) and very high resolution aerial images (136 Mpx of the ISPRS-EuroSDR benchmark). Frames extracted from videos of different quality are also available (ETH3D and Tanks and Temples).

Ground truth data for benchmarking MVS methods are usually acquired with laser scanner systems and used in the form of a point cloud. Strecha and Middlebury MVS convert the point cloud into a triangle mesh, yet the reference models are not publicly available from Middlebury MVS. In some cases, the ground truth is available only for “training” data, while additional scenes are provided for the evaluation (ETH3D and Tanks and Temples).

Most of the evaluation procedures impose a resampling or regularization of the MVS 3D data to be evaluated and, if the submitted result is a mesh, a conversion into point cloud (DTU Robot Image Data Sets, ETH3D, Tanks and Temples) is performed.

The investigated methods are often evaluated by submitting the obtained results online (e.g., Middlebury MVS and 3DOMcity); however, open source code is also made available for offline testing and training (DTU Robot Image Data Sets, ETH3D and Tanks and Temples).

The assessment protocol requires the reference/ground truth (GT) and tested/submitted data (D) to be aligned, i.e., co-registered in the same reference system. This may be accomplished in different ways: (i) using the provided image interior and exterior orientation parameters (Middlebury MVS and ETH3D); (ii) computing a 7-Degrees of Freedom (DoF) spatial transformation through absolute orientation of the image exterior orientation parameters (3DOMcity and Tanks and Temples); and (iii) with an iterative closest point (ICP) refinement between the reference and test data (Tanks and Temples).

The common metrics used in the evaluation are accuracy and completeness (Middlebury MVS, DTU Robot Image Data Set and 3DOMcity), also defined, respectively, as precision and recall (ETH3D and Tanks and Temples). Both criteria entail the computation of the distance between the two models. For the accuracy assessment the distance is computed from the submitted data (D) to the ground truth (GT). For the completeness evaluation, it is the opposite, i.e., from GT to D. The computed distances can be signed (Middlebury MVS and 3DOMcity) or unsigned (Tanks and Temples). A threshold distance is usually adopted to find the fraction or percentage of points falling within the allowable threshold, which is decided according to the data density and noise. As additional accuracy parameters, DTU Robot Image Data Set and 3DOMcity characterize the distance distributions with statistics, such as the mean and median values, also performing some outlier removal. ETH3D and Tanks and Temples combine the accuracy/precision p and completeness/recall r values into a single score, i.e., their harmonic mean (*F1* in ETH3D and *F* in Tanks and Temples), computed as: (*2∙p∙r*)/(*p + r*).

### 3.2. Surface Reconstruction and Assessment Criteria

Although aiming at the quality assessment of MVS approaches, the benchmarks described in the previous section mainly focus on dense point clouds. However, the surface reconstruction problem is also relevant in computer graphics. A survey on surface reconstruction methods from point clouds in computer graphics was provided by [52], who also distinguished the different evaluation criteria in geometric accuracy, topological accuracy, structure recovery and reproducibility. An analogy can be established between the quality assessment in photogrammetry and computer vision (Section 3.1) and the geometric accuracy in this context, which also requires the comparison with a ground truth. The Hausdorff distance (i.e., the maximum of the distances of all points of one mesh to the other [53]), mean and root mean square distance [54] or error in normals are frequently used geometric error measures. Metro [55] is a very popular tool for measuring the (geometric) difference between a reference mesh and its simplified version.

**Figure 2:**
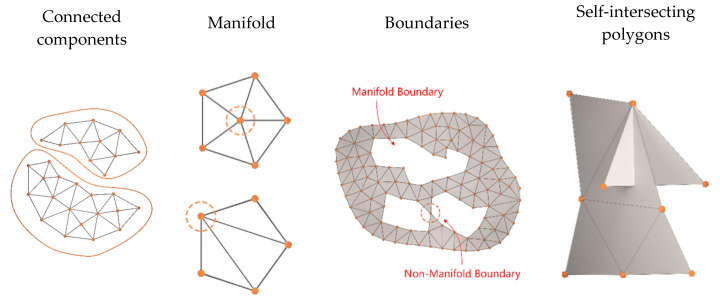
Examples of polygonal mesh topology properties.

When dealing with polygonal mesh surfaces, while geometry mainly refers to the position of vertices, topology refers to the connectivity, or graph, of the mesh elements, i.e., vertices, edges and triangles [56]. Visual exemplifications of connected components, manifolds, self-intersections and boundaries are shown in Figure 2.

With the aim of overcoming the issue of a “real” ground truth, i.e., the lack of the computational representation of the reference surface, Berger et al. [57] proposed a benchmark for the assessment of surface reconstruction algorithms from point clouds where point cloud data are simulated as being acquired with a laser scanner. Implicit surfaces, i.e., continuous and smoothly blended surfaces [58] of different complexity, are used as initial geometric shapes for sampling and are adopted as reference models for a quantitative evaluation. Both geometric and topological error measures are reported.

Crucial in computer graphics is the assessment of the mesh quality degradation resulting from simplification, resampling and other operations, for example compression and watermarking, which alter the original mesh not only from a geometric/topological point of view but also introduce visual distortions. Mesh visual quality (MVQ) assessment is, indeed, adopted as a criterion to design and optimize mesh processing algorithms [59]. While in photogrammetry the quality of a produced model is usually assessed in terms of its accuracy, precision and resolution, in computer graphics the term quality indicates “*the visual impact of the artefacts introduced by computer graphics*” algorithms [60]. It has been shown that metrics frequently adopted for assessing the geometric accuracy of the mesh (i.e., Hausdorff, mean and root mean square distances) do not correlate well with the human perception of surface quality and therefore quality scores consistent with the perception of human observers have been introduced [60,61,62]. These perceptually driven quality metrics, which try to mimic the human visual system (HVS), are based on roughness, local curvature, saliency, contrast and structural properties of the mesh and require a reference mesh to estimate the introduced degradation. Roughness infers the geometric non-smoothness [63] of a surface and can be computed as either a local or a global property: while local roughness may provide the high frequency behavior of the mesh vertices in a local region, global roughness is an indication of the average low frequency surface characteristic [64]. Curvature is also adopted as a measure to indicate structure and noise, well correlating with visual experience [65]. Databases comprising reference and distorted meshes are publicly available for assessing MVQ degradation introduced by geometrical processing [66] (subjective quality assessment of 3D models). However, since MVQ metrics are computationally expensive, the 3D models contained in these databases are of lower resolution (up to 50k vertices and 100k triangles) than typical models for photogrammetric applications. Lately, machine learning tools have been applied to estimate visual saliency and roughness of meshes without any reference model [67].

## 4. Investigated Surface Generation Methods

Three surface generation methods are hereafter considered. All three approaches require images with known camera parameters (interior and exterior) as input. Therefore, to avoid bias, all three methods share the same interior and exterior camera parameters and undistorted images, as well as depth values and dense point clouds estimations.

SIFT feature points [68] are first extracted, then matched with cascade hashing [69] and finally the camera poses are computed along with the sparse point cloud within an incremental bundle adjustment, as implemented within OpenMVG library [70]. Distortions are removed from the images before the dense matching step, which generates depth maps and a dense point cloud. Based on PatchMatch stereo [71,72], Shen [24] introduced a patch-based stereo approach where the depth of each pixel is calculated using random assignment and spatial propagation. OpenMVS is an open-source library that closely follows this idea while applying some optimization steps for more efficiency and is thus broadly used in 3D reconstruction research [42,73,74]. First, the best neighboring views are selected based on viewing direction criteria, and potential stereo pairs are formed. Rough depth maps are generated based on the sparse point clouds and iteratively refined using photo-consistency (zero mean normalized cross correlation ZNCC). Estimated depth maps are subsequently filtered taking into consideration visibility constraints while enforcing consistency among neighboring views. Finally, overlapping depth maps are merged to generate the fused dense 3D point cloud of the scene by minimizing redundancies and eliminating occluded areas.

The three employed surface generation methods are reported in detail in the following sections.

### 4.1. Photo-Consistent Volume Integration and Mesh Refinement (Method 1, M1)

The mesh reconstruction method exploiting photo-consistency and image visibility information is based on the approach introduced by Jancosek and Pajdla [13] as implemented by OpenMVS. The 3D space is initially discretized in tetrahedra using Delaunay tetrahedralization starting from the dense points and free space is modeled from the visibility information of the input 3D points. The final surface results as the interface between the free and the full space (graph cut optimization) while respecting visibility constraints, i.e., the image orientation and the projection of the 3D points back to the 2D image plane. Several mesh optimization steps can be performed to obtain an optimal mesh result, being pure geometric, such as smoothing, non-manifold and spike removal, or photo-consistent. Surface curvature, as expressed by point normals, is also taken into consideration during mesh reconstruction and thus complex regions are represented with high density triangles, while smoother areas may be wrapped into triangles of larger edges [75]. Photo-consistent refinement algorithms are generally efficient enough to produce detailed surfaces even from a rough input. In this method, an extra step of mesh refinement solution based on the idea described in [16] is implemented, by adding a photometric consistency score along with the geometric regularization term weighted by a scalar regularization weight. Mesh texturing is also enabled in this method, by assigning a best view to each face and generating a texture atlas, as described in [76]. In our experiments, following the OpenMVS implementation, we performed the extra mesh refinement step in order to take full advantage of the visibility information.

### 4.2. Surface Generation from Point Cloud (Method 2, M2)

Poisson surface reconstruction from oriented (i.e., with normals) point clouds [14] is a well-known and commonly adopted meshing approach. It creates a watertight surface, solving the reconstruction problem as a solution of a Poisson equation. An indicator function is computed with value one inside the surface and zero outside. In this work, M2 is based on the screened Poisson formulation [77], as implemented in CloudCompare [78]. The term “to screen” is adopted by the authors to indicate the screening term associated with the Poisson equation. The screening term reduces the over-smoothing of the data by introducing a soft constraint that forces the reconstruction to follow the input points. The volume occupied by the orientated points is partitioned using an adaptive octree structure, whose depth d (or level) can be decided accordingly. Selecting a depth implies constructing a voxel grid with a resolution no better than 2^d^ × 2^d^ × 2^d^. The octree level is automatically adapted to the original point sampling density, with the selected reconstruction depth being an indication of the maximum achievable mesh resolution. Beside the depth value, another critical parameter is the samples per node. It defines the number of points included in each voxel grid or node: the more noisy are the input data, the higher should be the number of points falling in each node of the octree, which may result in a loss of geometric details. If the original points have color information, as in our experiments, the RGB values are interpolated and transferred to the vertices of the generated mesh.

### 4.3. TSDF Volume Integration (Method 3, M3)

The Truncated Signed Distance Field (TSDF) volume integration is a volumetric reconstruction method broadly used while working with low-cost RGB-D sensors and real-time scenarios. It became a standard method since Newcombe et al. [15] used it in the KinectFusion project followed by various extensions and optimizations thereafter [79,80,81,82]. TSDF methods divide the 3D space (volume) into a discretized set of voxels and fuse distance information into them and is optimized for reconstruction speed. It is commonly combined with the marching cubes algorithm [83] (to generate a mesh, using the voxel grid created by TSDF and creating triangles on the edges. In more detail, SDF functions yield the shortest distance to any surface for every 3D point: depending on the sign, a point can be inside (negative) or outside (positive) the object boundaries, with the surface boundaries lying exactly on the zero crossing. On the other hand, in TSDF methods, a truncation threshold is added to omit everything outside this range. The standard method, although efficient under certain scenarios, has some default fundamental limitations as the voxel size itself defines the resolution of the final mesh and anything below this threshold cannot be reconstructed or erroneous results are produced when slanted surfaces are present, requiring alternative optimization solutions (e.g., [82,84]). In this work, M3 uses the TSDF implemented in the Intel Open3D library [19]. The resulting mesh may consist of a large number of unnecessary polygons, so further optimization steps may be performed: (1) merge the vertices of the mesh that are within a certain tolerance; (2) eliminate all edges and vertices that are non-manifold; (3) divide the mesh into clusters; and (4) eliminate all clusters with an area less than a certain value.

## 5. Datasets and Evaluation Metrics

The purpose of this study, namely the understanding and quantification of the potential benefits of surface reconstruction methods fully integrated into the photogrammetric pipeline, requires the usage of available benchmark data and metrics (Section 3), while pushing towards the identification of additional test cases and evaluation measures.

### 5.1. Datasets

Covering a broad range of application scenarios was the highest priority while choosing the evaluation datasets summarized in Table 1. Some of them are derived from existing benchmarks while others originate from original projects realized by FBK/3DOM.

Table 2 reports the selected parameters for the three investigated methods and the obtained final mesh resolution.

### 5.2. Evaluation Approach and Criteria

To enable the evaluation approach, a series of steps was undertaken. First, for the datasets for which the ground truth is available in the form of a point cloud, a surface was reconstructed using the same approach as in Section 4.2, preserving the original point cloud resolution. The meshing result was evaluated by computing the distance between the original point cloud and the derived mesh. Only the vertices that fall within a defined threshold (i.e., three times the average point cloud resolution) were retained. Moreover, interpolated triangles with a side length greater than about ten times the average mesh resolution and small disconnected components were eliminated from the mesh models generated by the three methods described in Section 4. Finally, a common datum was defined for the reference and evaluated meshes. The co-registration between the reference mesh and surfaces to be compared (called “data”) was performed in a two-step procedure: (i) an absolute orientation through reference points or image exterior orientation parameters where available; and (ii) 7-DoF spatial transformation refinement through ICP between the photogrammetric dense point cloud and the reference mesh.

The following metrics were used to evaluate the results:

- *Accuracy* was evaluated as the signed Euclidean distance between the vertices of the (photogrammetric) data mesh and the (scanner) reference mesh. The signed Euclidean distance was chosen instead of the Hausdorff distance to highlight any possible systematic error. For this, both CloudCompare and Meshlab [85] implementations were tested, providing equivalent results. The following values were computed: mean, standard deviation (STDV), median and normalized maximum absolute deviation from the median (NMAD = 1.4826 × MAD), root mean square (RMS) and outliers percentage.
- *Completeness* was defined as the signed Euclidean distance between the (scanner) reference mesh and the (photogrammetric) data mesh. The percentage of vertices of photogrammetric data mesh falling within the defined threshold (in%) was adopted as a measure for completeness.
- *F-score* was defined as in [42] (see Section 3.1).
- Local *roughness* was computed as the absolute distance between the mesh vertex and the best fitting plane estimated on its nearest neighbors within a defined kernel size. The method implemented in CloudCompare was adopted. Adapting the standard parameters generally used to quantify the roughness [86], mean and RMS roughness values are reported to describe the local behavior of the vertices in their local region (i.e., within the selected kernel). The kernel size was carefully selected according to surface resolution.
- Local *noise* was assessed on selected planar regions where the plane fitting RMS was computed.
- *Sections* were extracted from the meshes and the mean and RMS signed distance values from data to reference are reported.
- Local *curvature variation*, expressed as normal change rate, was computed over a kernel size, i.e., the radius defining the neighbor vertices around each point where the curvature was estimated. As for the roughness metric, the kernel size was decided according to the surface resolution and size of the geometric elements (3D edges). The normal change rate is shown as a color map to highlight high geometric details (e.g., 3D edges), which appear as sharp green to red contours, and high frequency noise, shown as scattered green to red areas. The method implemented in CloudCompare was here adopted.
- The *topology* of each generated surface is evaluated in terms of the percentage of self-intersecting triangles over the total number of faces.

Given the above, the accuracy, completeness and F-score provide insight on the global geometric correctness of the reconstructed mesh, or in other words its closeness to the reference model. At the same time, the roughness and fitting of planar areas are a measure of the high frequency noise generated in the meshing process, while the normal change rate mainly shows the ability of reproducing geometric elements, such as 3D edges and contours. Finally, the percentage of self-intersecting triangles is an indication of the level of topological errors produced by the surface generation approach.

## 6. Results and Discussion

In this section, the results of the performed analyses are discussed. Firstly, the dataset without ground truth data is presented, reporting evaluations in terms of profiles, normal change rate maps and plane fitting (Section 6.1). Then, mesh results with datasets featuring a ground truth mesh are presented (Section 6.2).

### 6.1. Evaluation without a Reference Mesh: The Aerial Case Study

**Figure 3:**
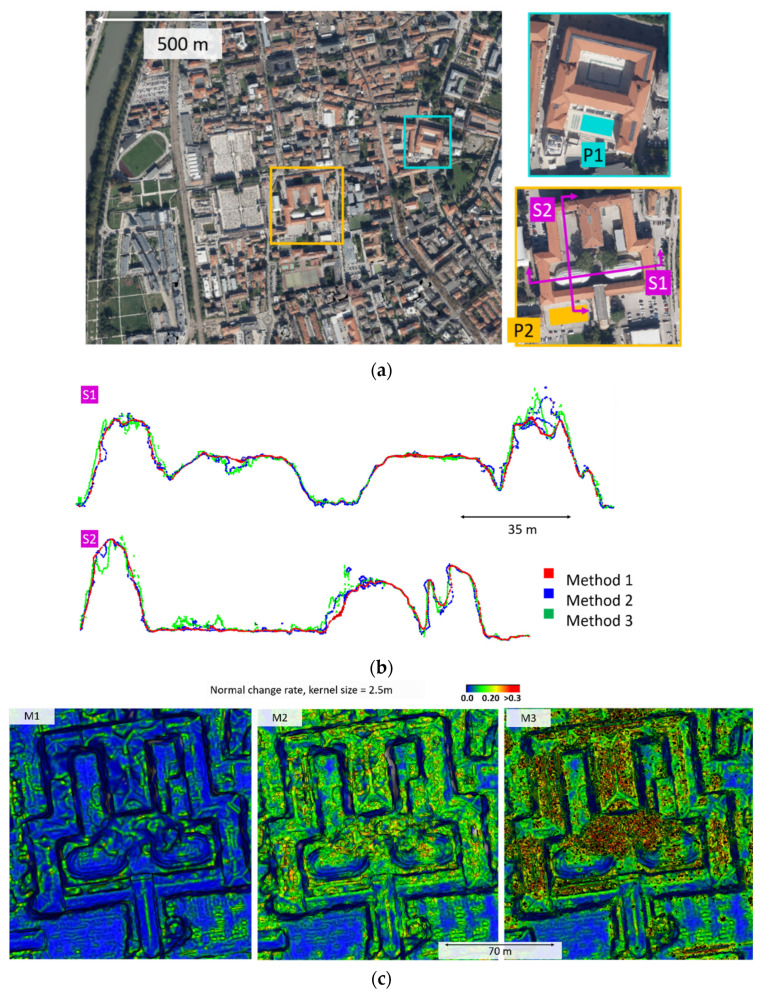
(**a**) Orthographic view of the urban area, with details of the extracted areas for the plane fitting analysis (P1 and P2) and sections (S1 and S2); (**b**) profiles of the extracted sections; and (**c**) normal change rate maps on a building.

For the aerial dataset, no reference model is available. Thus, the quantitative evaluation is reported in terms of plane fitting RMS in two different areas, P1 and P2 in Figure 3a. The difference in the surface reconstruction approaches is also qualitatively shown in the section profiles S1 and S2 (Figure 3b) and normal rate change (Figure 3c).

High noise and discrepancies can be observed while comparing the three methods. All methods present topological errors, with non-manifold vertices as well as self-intersecting faces for M2 and M3. M1 appears less noisy, in terms of both plane fitting RMS (Table 3) and normal change rate (Figure 3c). The section profiles also show a less bumpy pattern.

**Figure 4:**
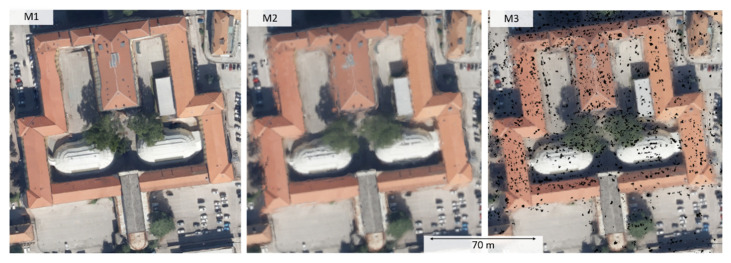
Orthographic view of the textured mesh from M1 and color vertex surfaces from M2 and M3.

To evaluate the suitability of the investigated approaches for specific photogrammetric applications that require orthophotos as the final outcome, orthographic views of the mesh models are shown (Figure 4). M1, which integrates the texturing step downstream the photogrammetric pipeline, provides a result visually more comparable to a standard orthophoto. The visual appearance is qualitatively better than the other two views, which are derived from color-vertex meshes. However, artifacts in the building edges due to geometric defects in the mesh model can be observed.

### 6.2. Evaluation with a Reference Mesh

**Figure 5:**
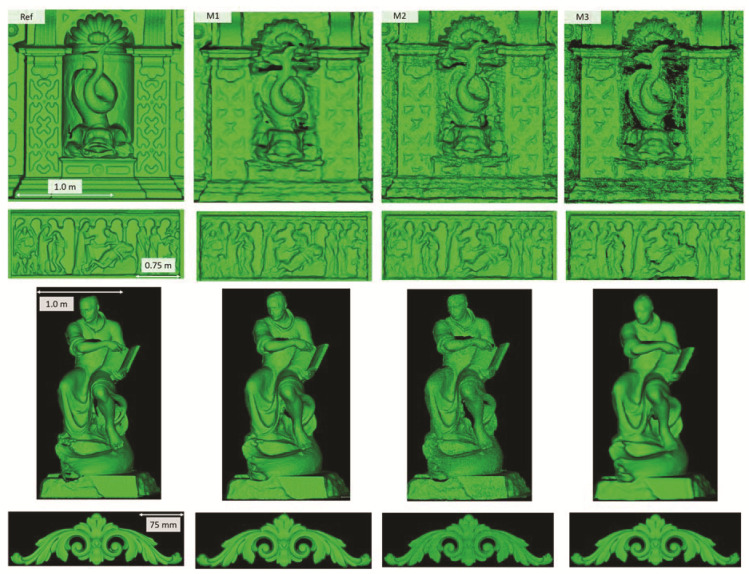
Shaded surface models of evaluated datasets. From left to right: Reference, M1, M2 and M3. From top to bottom: *Fountain*, *Modena’s* bas-relief, *Ignatius* and *Wooden ornament*.

**Figure 6:**
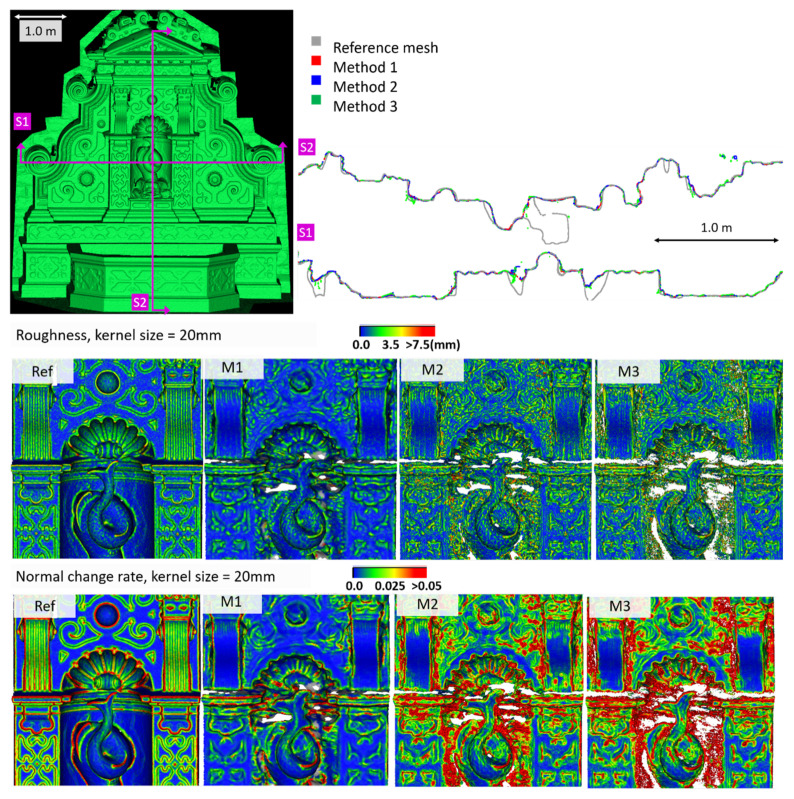
Section profiles (**top**); roughness (**middle**); and normal change rate maps (**bottom**) for the *Fountain* dataset.

**Figure 7:**
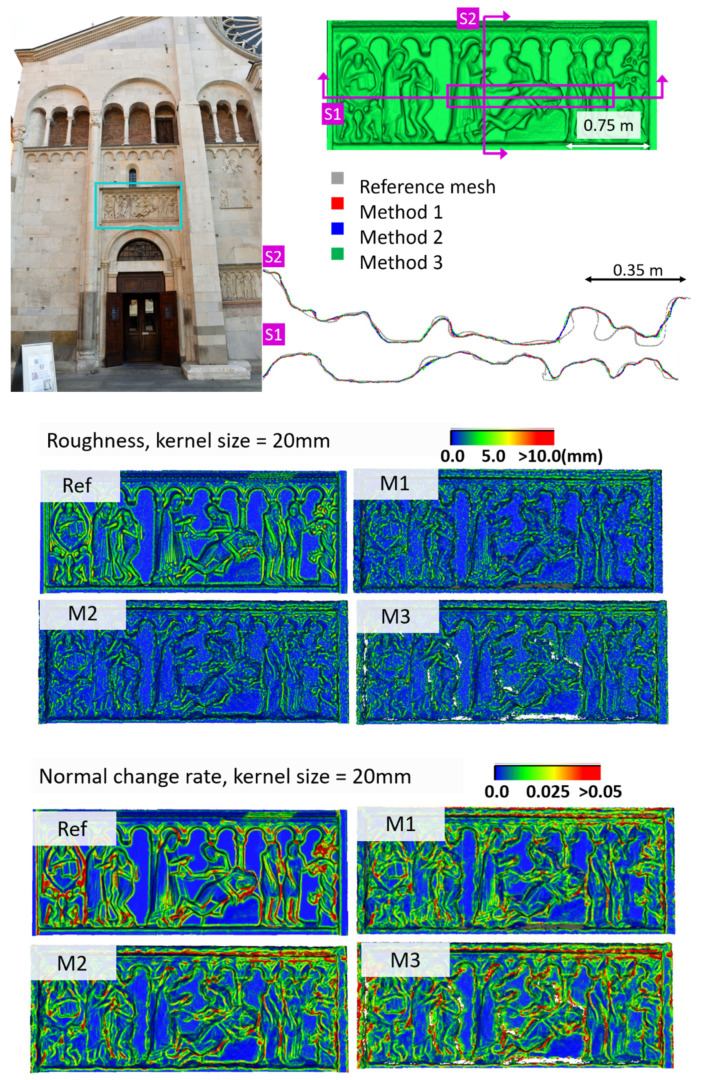
Section profiles (**top**); roughness (**middle**); and normal change rate maps (**bottom**) for the *Modena* dataset.

**Figure 8:**
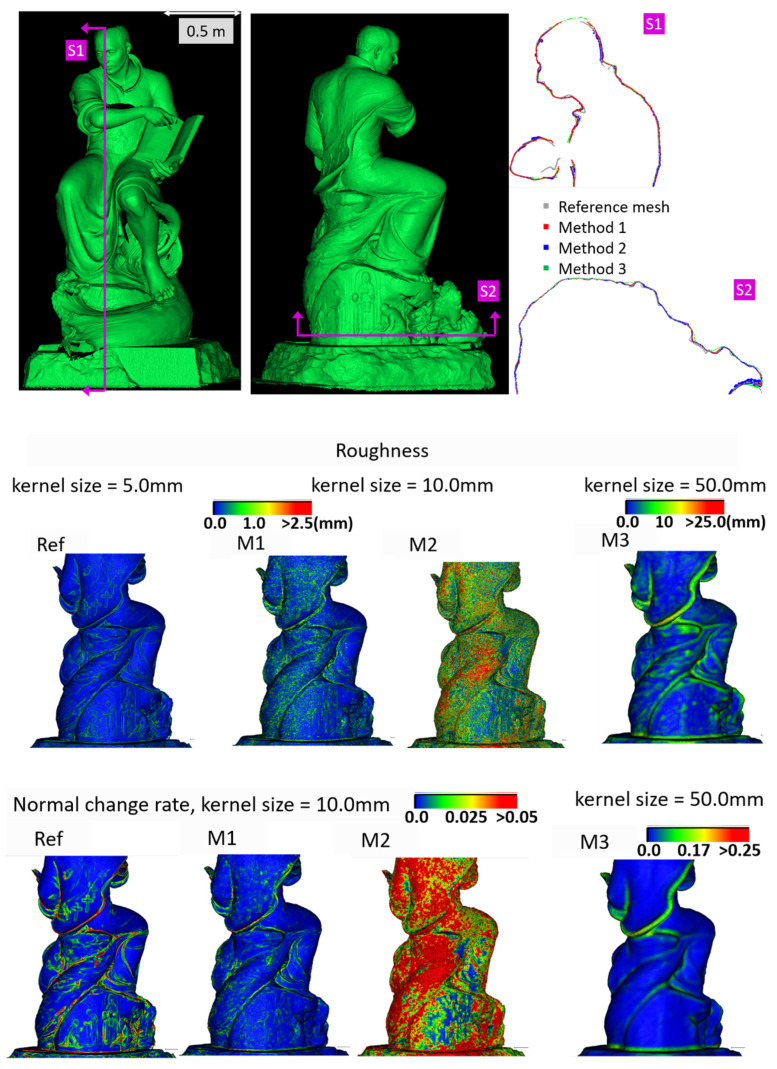
Section profiles (**top**); roughness (**middle**); and normal change rate maps (**bottom**) for the *Ignatius* dataset.

**Figure 9:**
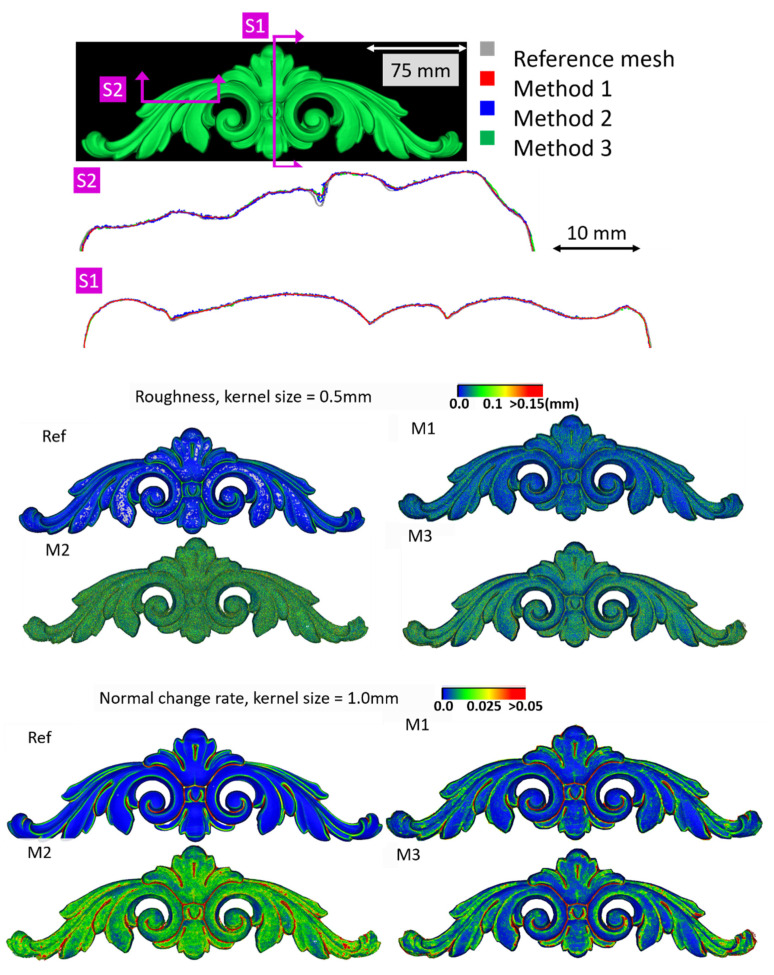
Section profiles (**top**); roughness (**middle**); and normal change rate maps (**bottom**) for the *Wooden ornament* dataset.

Figure 5 shows the surface models for the datasets where a reference surface model is available. The related analyses are summarized in Table 4, Table 5, Table 6 and Table 7 (see Section 5.2 for the definition of the metrics) and visually shown in Figure 6, Figure 7, Figure 8 and Figure 9 for the datasets *Fountain*, *Modena*, *Ignatius* and *Wooden ornament,* respectively. The roughness map provides information on the geometric non-smoothness of the surface. The normal change rate map highlights high geometric details (e.g., 3D edges), which appear in the images as sharp green to red contours, and high frequency noise, shown as scattered green to red areas.

The accuracy, completeness and F-score values (Table 4, Table 5, Table 6 and Table 7) reveal that the three investigated approaches perform similarly, with M1 and M2 usually outperforming M3.

M1 also exhibits the best metrics in terms of roughness for all the datasets. The visual inspection and metric values for the section profiles point out that all three methods tend to over-smooth the geometric details compared to the ground truth and that the sections from M1 are usually less noisy. All the investigated mesh models present non-manifold vertices, and, other than the *Modena*’s bas-relief (Table 5), M2 (Table 4, Table 6 and Table 7) and M3 (Table 4 and Table 7) are also characterized by self-intersecting faces.

The normal change rate maps convey additional insight on the different performances of the three surface generation methods. It is evident that none of the approaches can reproduce the geometric details of the reference mesh. However, M3 and especially M2 are more affected by high frequency noise, easily distinguishable in the green to red spots spread over the models (Figure 6 and Figure 8 from the *Fountain* and *Wooden ornament* datasets). For *Ignatius*, due to the significantly lower resolution of M3, a different kernel size is adopted for the normal change rate estimation, clearly implying an over-smoothed geometry with respect to the other approaches. In the *Modena*’s bas-relief, the normal change rate does not highlight significant differences among the investigated methods.

From the analysis of the roughness and normal change rate maps, it can be deduced that the methods do not show significant differences, when the starting data (dense point cloud or depth maps) are not heavily affected by high frequency noise (*Modena* dataset, Figure 7). When noise characterizes the intermediate MVS results such as for the *Fountain* (Figure 6), *Ignatius* (Figure 8) and *Wooden ornament* (Figure 9) datasets, M1 generally produces less noisy surfaces while preserving better the geometric details.

## 7. Conclusions

Surface or mesh reconstruction is a cross-disciplinary topic and an important step in a 3D modeling procedure. It can be fully integrated into the image-based pipeline as the final output of the MVS step or applied separately from the main workflow, which implies the use of popular surface reconstruction algorithms such as Poisson.

We investigated three different approaches of surface reconstruction in the context of photogrammetric applications: (1) the mesh generation step, incorporated in the reconstruction pipeline, takes into account photo-consistency and visibility information (M1); (2) the surface reconstruction is “outsourced” from the main reconstruction workflow and does not exploit visibility constraints or photo-consistency checks (M2); and (3) provided the image orientation parameters, the mesh is generated by integrating the depth maps with a volumetric method, without any visibility or photo-consistency information (M3). The comparative analysis aimed at quantifying the improvement of approaches fully integrated into the 3D reconstruction procedure and leveraging geometric and photo-consistency constraints, against methods disjointed from the dense reconstruction procedure that do not further exploit image content information or the results from bundle adjustment.

We first revised the concepts and steps of MVS and reviewed existing benchmarks, highlighting their limitations in the context of this work. Many of the publicly available data do not provide reference data in the form of mesh models and the employed assessment criteria are usually narrowed to the global geometric correctness through accuracy and completeness scores, ignoring other important features such as the reproduction of fine geometric details or noise level. An overview of surface assessment criteria adopted in computer graphics was also provided, with a focus on those considered in this study to quantify the reconstruction noise and geometric details.

The three considered methods were introduced, and the selected datasets and evaluation metrics were described. Drawing a definite conclusion was out of the scope of the paper. The results of the investigation show that, in experiments with a reference model, M1 and M2 performed similarly in terms of accuracy and completeness. However, the surface generation method integrated into the image-based reconstruction workflow (M1) generally outperformed the other two approaches in recovering geometric details and reducing the noise in all the considered case studies, regardless of the characteristics of the given images (scale, resolution, texture, etc.).

Although relevant for some applications, especially in real time, computational efficiency was not included in this evaluation, because the main interest was to test the best achievable quality even at the expense of long calculation times. However, it should be mentioned that M3 proved to be computationally more efficient than M1 and M2, i.e., on average 5–10 times faster than M1 and 2.5–5 time faster than M2, differences that can get larger as the complexity of the dataset increases in terms of resolution and noise. The presented study also emphasized the lack of benchmarks and assessment criteria specifically addressing the surface reconstruction problem for applications where metric accuracy matters.

Our future work will include the expansion of the current investigation to further MVS approaches and integrating perceptual evaluation metrics into rigorous accuracy assessment procedures. The robustness of the different methods to possible variations in the interior and exterior orientation parameters will also be examined. Moreover, we plan to further investigate the inclusion of visibility and semantic constraints in the 3D reconstruction pipeline towards the optimization of the final products.

## Figures and Tables

**Table 1:** Case studies and related characteristics.

Dataset	Type of Scene	Type of Acquisition	Num of Images/Total Mpx	Scene Size/Mean Image GSD	Ground Truth	Evaluation Criteria
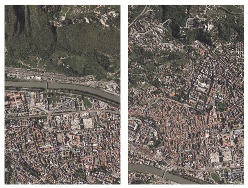				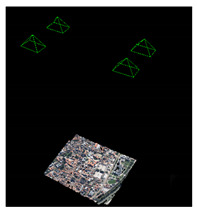		
FBK/AVT	Urban	Aerial nadir—single shots	4/120	(1 × 1 × 0.1) km^3^/10 cm	-	Profiles, plane fitting, topology correctness
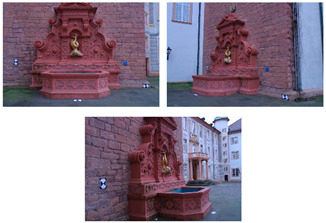				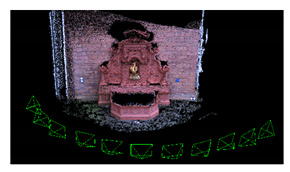		
Strecha Fountain	Building facade	Terrestrial—single shots	11/66	(5 × 4 × 5) m^3^/30 mm	Mesh from laser scanner	Accuracy, completeness, roughness, profiles, topology correctness
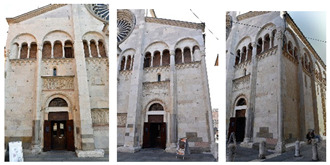				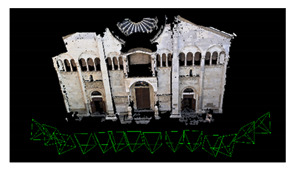		
FBK/3DOModena	Building facade	Terrestrial—single shots	14/320	(11 × 3 × 9) m^3^/20 mm	Mesh from laser scanner	Accuracy, completeness, roughness, profiles, topology correctness
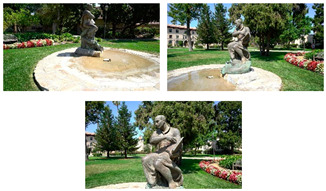				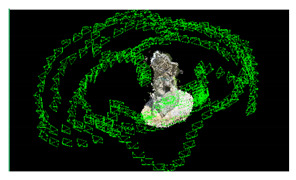		
Tanks and Temples—Ignatius	Statue	Terrestrial—video	263/535	(2 × 2 × 3) m^3^/30 mm	Mesh from laser scanner	Accuracy, completeness, roughness, profiles, topology correctness
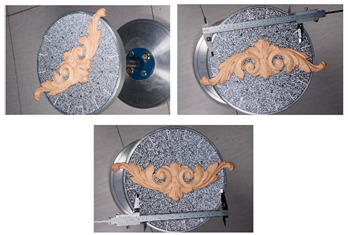				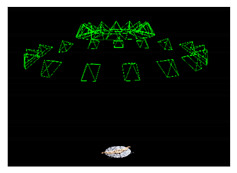		
FBK/3DOM wooden ornament	Asset	Terrestrial—single shots	32/740	(305 × 95 × 25) mm^3^/0.6 mm	Mesh from structured light scanner	Accuracy, completeness, roughness, profiles, topology correctness

**Table 2:** Main parameters and final mesh resolution of the investigated methods.

		FBK/AVT	Strecha Fountain	Tanks and Temples—Ignatius	FBK/3DOM Modena	FBK/3DOM Wooden Ornament
Image Resolution for Depth Maps and Dense Point Cloud Generation		¼	Full	Full	¼	Full
M1	Regularity weight	0.4	0.4	0.4	0.4	0.4
	Resolution (mm)	80.0	1.3	1.2	3.0	0.04
M2	Voxel grid size (mm)	160.0	0.7	0.3	1.5	0.02
	Samples per node	20	1.5	20	1.5	20
	Resolution (mm)	160.0	0.3	0.2	0.7	0.01
M3	Voxel grid size (mm)	500.0	3.8	15	6.2	1.4
	Resolution (mm)	160.0	2.7	5.0	3.0	0.07

**Table 3:** Quantitative and topological analyses for the aerial dataset: plane fitting and percentage of self-intersecting faces.

Method	Plane Fitting RMS (m)		Percent of Self-Intersecting Faces
	P1	P2	
M1	**0.352**	**0.602**	-
M2	0.391	0.606	0.01%
M3	0.385	0.547	0.5%

**Table 4:** Quantitative and topological analyses for the *Fountain* dataset. Values are in mm. Threshold and kernel values are set equal to 9.0 and 10 mm, respectively. Mean and RMS values in the Sections columns are double as we considered two sections (Figure 6).

Method	Accuracy						Completeness	F-Score	Roughness		Sections		% of Self-Intersecting Faces
	MEAN	STDV	RMS	MEDIAN	NMAD	OUT%	IN%		MEAN	RMS	MEAN	RMS	
M1	3.3	**11.0**	**11.5**	1.7	6.8	**3.7**	**73.4**	0.886	**1.0**	**1.3**	**6.5**	**14.9**	-
											0.4	15.9	
M2	**2.1**	12.6	12.7	**0.4**	**6.7**	4.1	73.6	**0.898**	1.7	2.2	6.9	16.2	0.03%
											**0.1**	**15.0**	
M3	3.6	27.9	28.1	2.1	8.6	7.8	71.1	0.827	1.8	2.3	8.7	18.3	0.07%
											−0.2	27.7	

**Table 5:** Quantitative and topological analyses for the *Modena* dataset. Values are in mm. Threshold and kernel values are set equal to 4.5 and 20 mm, respectively. Mean and RMS values in the Sections columns are double as we considered two sections (Figure 7).

Method	Accuracy						Completeness	F-Score	Roughness		Sections		% of Self-Intersecting Faces
	MEAN	STDV	RMS	MEDIAN	NMAD	OUT%	IN%		MEAN	RMS	MEAN	RMS	
M1	5.4	21.2	21.9	**−0.1**	**5.6**	9.8	**54.3**	**0.779**	**1.0**	**1.4**	**−0.1**	**6.0**	-
											**1.3**	**8.4**	
M2	**5.0**	**17.5**	**18.2**	0.8	6.4	**7.5**	52.7	0.758	1.5	2.0	1.0	8.0	-
											2.0	9.0	
M3	7.1	20.9	22.1	1.3	7.1	9.1	52.7	0.736	2.1	2.8	0.9	7.2	-
											1.7	9.0	

**Table 6:** Quantitative and topological analyses for the *Ignatius* dataset. Values are in mm. Threshold and kernel values are set equal to 3 and 10 mm, respectively. Mean and RMS values in the Sections columns are double as we considered two sections (Figure 8).

Method	Accuracy						Completeness	F-Score	Roughness		Sections		% of Self-Intersecting Faces
	MEAN	STDV	RMS	MEDIAN	NMAD	OUT%	IN%		MEAN	RMS	MEAN	RMS	
M1	**1.6**	**3.2**	**3.6**	1.4	**2.7**	**1.5**	65.3	**0.820**	**0.4**	**0.5**	3.4	**7.3**	-
											0.5	**2.7**	
M2	1.7	7.4	7.6	**1.0**	3.0	2.7	**85.7**	0.798	1.2	1.5	**2.5**	7.8	0.02%
											**−0.2**	3.1	
M3	4.9	11.4	12.4	3.0	3.0	9.2	58.8	0.643	3.6 *	3.3 *	5.4	9.7	-
											2.4	4.3	

* Kernel size equal to 50 mm.

**Table 7:** Quantitative and topological analyses for the *Wooden ornament* dataset. Values are in mm. Threshold and kernel values are set equal to 0.225 and 0.5 mm, respectively. Mean and RMS values in the Sections columns are double as we considered two sections (Figure 9).

Method	Accuracy						Completeness	F-Score	Roughness		Sections		% of Self-Intersecting Faces
	MEAN	STDV	RMS	MEDIAN	NMAD	OUT%	IN%		MEAN	RMS	MEAN	RMS	
M1	0.06	0.23	0.24	**0.03**	**0.11**	5.5	**99.9**	0.80	**0.02**	**0.03**	0.05	**0.12**	-
											**0.05**	0.14	
M2	**0.05**	**0.13**	**0.14**	0.04	0.11	**1.9**	90.8	**0.93**	0.06	0.07	**0.04**	0.14	0.002%
											0.07	**0.10**	
M3	0.36	1.34	1.4	0.03	0.14	10.1	77.9	0.86	0.04	0.05	0.09	0.17	0.06%
											0.61	0.13	
